# Changes in the sympathetic innervation of the gut in rotenone treated mice as possible early biomarker for Parkinson’s disease

**DOI:** 10.1007/s10286-016-0358-6

**Published:** 2016-05-13

**Authors:** Mike Arnhold, Yanina Dening, Michaël Chopin, Esteban Arévalo, Mathias Schwarz, Heinz Reichmann, Gabriele Gille, Richard H. W. Funk, Francisco Pan-Montojo

**Affiliations:** Neurologische Klinik und Poliklinik, Klinikum der Universität München, Marchioninistr. 15, 81377 Munich, Germany; Munich Cluster for Systems Neurology, Munich, Germany; Institut für Anatomie, TU-Dresden, Fetscherstr. 74, 01307 Dresden, Germany; Klinik und Poliklinik für Neurologie, Uniklinikum Carl-Gustav Carus, Fetscherstr. 74, 01307 Dresden, Germany; Center for Regenerative Therapies Dresden, Tatzberg 47/49, 01307 Dresden, Germany; The Walter and Eliza Hall Institute, 1G Royal Parade, Parkville, VIC 3052 Australia; Department of Medical Biology, University of Melbourne, Parkville, VIC 3010 Australia

**Keywords:** Parkinson’s disease, Enteric sympathetic innervation, Constipation, alpha-synuclein pathology, Biomarker

## Abstract

**Introduction:**

Involvement of the peripheral nervous system (PNS) is relatively common in Parkinson’s disease (PD) patients. PNS alterations appear early in the course of the disease and are responsible for some of the non-motor symptoms observed in PD patients. In previous studies, we have shown that environmental toxins can trigger the disease by acting on the enteric nervous system.

**Material and methods:**

Here, we analyzed the effect of mitochondrial Complex I inhibition on sympathetic neuritis in vivo and sympathetic neurons in vitro. Combining in vivo imaging and protein expression profiling.

**Results:**

we found that rotenone, a widely used mitochondrial Complex I inhibitor decreases the density of sympathetic neurites innervating the gut in vivo, while in vitro, it induces the redistribution of intracellular alpha-synuclein and neurite degeneration. Interestingly, sympathetic neurons are much more resistant to rotenone exposure than mesencephalic dopaminergic neurons.

**Conclusion:**

Altogether, these results suggest that enteric sympathetic denervation could be an initial pre-motor alteration in PD progression that could be used as an early biomarker of the disease.

**Electronic supplementary material:**

The online version of this article (doi:10.1007/s10286-016-0358-6) contains supplementary material, which is available to authorized users.

## Introduction

Hallmark lesions of Parkinson’s disease (PD) were traditionally considered to be present in the dopaminergic neurons of the substantia nigra (SN) and in the noradrenergic neurons of the locus coeruleus (LC). However, pathological studies showed that typically PD patients have lesions in other CNS and peripheral nervous system (PNS) structures [e.g. the enteric nervous system (ENS), the sympathetic CG, the IML of the spinal cord, the motor nucleus of the vagus or the amygdale] [1, 2]. These lesions are not exclusive for PD [3] and mainly consist in intraneuronal and intraglial alpha-synuclein aggregates called Lewy bodies (LB) and Lewy neurites (LN).

LBs in the sympathetic ganglia were first observed by Herzog and colleagues [4]. Although alpha-synuclein is the main constituent of LB, many more molecules have been identified in these structures. Among these are the tau protein, advance glycation end-products (AGES), heat-shock proteins, microtubule associated proteins (MAP), neurofilaments, synphilins or tyrosine hydroxylase (TH) [5]. In the ganglia, LBs occur mainly in the nerve cell processes and the majority of LB-containing processes are unmyelinated axons [6]. Paravertebral and celiac sympathetic ganglia are the predilection sites for LBs and no LBs are found in the dorsal root ganglia [7]. In idiopathic PD most of the patients that show PD related inclusions in CNS sites already present LB and LN in the sympathetic ganglia [8]. Based on autopsies performed on PD patients and healthy individuals, Braak and colleagues [9] developed a pathological staging of the disease. According to this staging, PD lesions follow an spatio-temporal pattern that starts in the olfactory bulb (OB) and the ENS progressing into the CNS through synaptically connected structures. This pathological staging of the disease seems to correlate well with the appearance of early non-motor symptoms in PD patients. These include hyposmia, gastrointestinal alterations, autonomic dysfunction and the experience of pain [10].

The degenerative process of the sympathetic ganglia has been observed in the cardiac sympathetic plexus in PD and Lewy body disease patients and classified within three stages in which neurite loss precedes neuronal loss [11, 12]. In the early stage, a few LBs are observed in the ganglia but the number of neurons and immunoreactivity for TH are well preserved. In the middle stage, many LBs are found in the ganglia and the number of neurons appears to be normal with H&E staining. However, a significant number of neuronal somata (about 20–30 % of neurons) are TH-immunonegative. In the advanced stage, there is apparent neuronal loss in the ganglia and the number of LBs is decreased compared with the middle stage. In a study using 6-^18^F-fluorodopamine PET–CT imaging, it was shown that this radiotracer was useful to detect sympathetic denervation of the heart, the thyroid gland and the renal cortex in PD patients with orthostatic hypotension [13].

In our previous studies, we have shown that orally administered rotenone, a pesticide inhibiting mitochondrial Complex I, induces alpha-synuclein accumulation in most of the regions described in Braak’s staging [14, 15]. The appearance of these alterations followed a spatio-temporal pattern similar to the one predicted in this staging and the resection of the nerves connecting the gut to the SNC (i.e. the vagus and sympathetic nerves) prevented the progression of the pathology.

In this study we analyzed whether the local effect of rotenone on the ENS in vivo would lead to alterations in its sympathetic innervation with similar morphological and structural alterations as the ones observed in PD patients. Additionally, we also analyzed and compared the effect of rotenone on dopaminergic and sympathetic neurons in vitro.

## Materials and methods

### Animal model

#### Animal housing

8-week-old C57/BL6J mice (Janvier, France) were housed at room temperature (RT) under a 12-h light/dark cycle. Food and water was provided ad libidum. All animal experiments were carried out in accordance with the National Institutes of Health Guide for the Care and Use of Laboratory Animals, and protocols were approved by the Saxonian Committee for Animal Research in Germany.

#### Oral rotenone administration

6-week-old mice were divided into two groups (*n* = 10) and treated 5 days a week for 4 months. A 1.2 mm × 60 mm gavage needle (Unimed, Switzerland) was used to administer 0.01 ml/g animal weight of rotenone solution corresponding to a 5 mg/kg dose. Controls were treated only with the vehicle solution [2 % carboxymethylcellulose (Sigma-Aldrich, Germany) and 1.25 % chloroform (Carl Roth, Germany)].

#### 1 h Stool collection test

This test was performed every month during treatment. Mice were placed on individual cages for 1 h. During this hour all pellets in the cage were collected and weighted.

#### Tissue preparation for histology and immunohistochemistry

Mice were killed with an overdose of ketamine and perfused intracardially first with 0.01 % heparin in phosphate buffer solution (PBS) and then with 4 % paraformaldehyde (PFA) in 0.1 M phosphate buffer (pH 7.4). After perfusion, the gastrointestinal tract and the coeliac ganglia were removed and kept in 4 % PFA for 24 h.

The muscle layer of the intestine was used for whole-mount immunostaining. The muscle layer was obtained post-fixation. In order to do this, intestine pieces were cut longitudinally and stretched like a sheet on a 4 % agarose gel with the muscle layer facing the gel. The mucosa was then ripped off with the help of fine straight forceps. The muscle layers were kept at −20 °C until use, in a freezing solution containing 50 % PBS, 25 % ethylene glycol and 25 % glycine.

#### Immunostaining procedures

Whole-mounts were stained using a freefloating immunostaining technique. Non-specific background staining was blocked overnight at 4 °C in BS. Sections were then incubated with sheep anti-TH(1:1,000, Pel-Freez, USA) and rabbit anti-ASYN (1:400, Santa-Cruz, USA) or with goat anti-ChAT (1:400, Chemicon, USA), chicken anti-βIII-tubulin (1:500, Novus Biologicals, USA) and rabbit anti-TH (1:1,000, Pel-Freez, USA) antibodies overnight at 4 °C, washed 4 × 15 min in PBS, incubated for 1 h at RT with the corresponding donkey anti-rabbit, anti-goat and anti-sheep (1:500) or donkey anti-chicken (1:200, Jackson Immunoresearch, USA) fluorescent secondary antibodies, washed 4 × 15 min in PBS again and mounted using Vectashield^®^ mounting medium for fluorescence with DAPI (Vector Laboratories, USA).

### Primary sympathetic neuronal cultures

#### Dissection and tissue disaggregation

Sympathetic neurons were isolated from postnatal (P2–P6) NMRI and C57J/BL6 mice as previously described [16]. Briefly, mice were placed on ice to cause hypothermia-induced anesthesia. Once anesthetized, mice were decapitated with a cut at the level of the heart under the arms and heads fixed with needles to a 4 % agarose gel. All procedures that followed were performed using a stereomicroscope (Zeiss, Germany, EU). Skin saliva glandules and muscle layers were removed to allow visualization of the sympathetic ganglia. Sympathetic superior ganglia are attached to the bifurcation of both carotid arteries. Therefore, sympathetic ganglia were normally removed together with the carotid artery and placed on PBS (Gibco, USA). Here, the sympathetic ganglion was freed from adjacent structures and placed in Hank’s buffer solution (HBSS) (Gibco, USA) on ice. Sympathetic ganglia from all mice were digested with 2.5 mg/ml collagenase in HBSS for 15–20 min (depending on the batch of collagenase and its enzymatic activity) followed by digestion in trypsin–EDTA for 10 min. After digestion, trypsin–EDTA was substituted by N2-Medium [Neurobasal-A (Invitrogen, Germany, EU), 1 % N2 supplement (Gibco, USA), 5 % fetal calf serum (FCS) (Sigma-Aldrich, Germany, EU), 0.2 % penicillin/streptomycin and 100 ng/ml neuronal growth factor (NGF) (BD-Bioscience, USA)]. Sympathetic ganglia were then mechanically dissociated using an insulin needle. The concentration of neurons per ml was calculated using a Neubauer^®^ cell counting chamber (Germany, EU). Briefly, 10 µl of neuron containing medium was placed on the Neubauer^®^ chamber and the number of sympathetic neurons was calculated as the mean of the number of neurons counted in four selected areas multiplied by 10,000.

#### Plating and cell culture maintenance

Sympathetic neurons were plated on collagen coated culture plates. Surface coating was performed under sterile conditions on the same day as the embryo preparation. Rat tail collagen (type I) (BD Bioscience, USA) was diluted using 0.02 N acetic acid to a concentration of 50 µg/ml. Around 200 µl of diluted collagen solution were pipette in every well and left at RT for 1 h. Wells were then washed 4 × 5 min using 500 µl PBS and left under the sterile hood until neurons were plated.

Neurons were plated on either 4-well culture plates (Nunc, Denmark, EU) or 15 µ-Slide 8 well ibiTreat chambers (Ibidi, Germany, EU) at a final concentration of 20,000 cells per 200 or 500 µl (depending on the well-plates used). One day after plating approximately 2/3 of the N2-Medium were substituted by Medium A (N2-Medium without FCS, 1 % B-27 supplement, 0.24 µg/ml 1-β-arabinofuranosylcytosine (Ara-C) and 0.2 % penicillin/streptomycin (Sigma-Aldrich, Germany, EU). On DIV3, all medium was completely substituted by Medium A. Medium was then changed every 2 days until DIV7.

#### Rotenone treatment in vitro

Rotenone treatment began on DIV7. Rotenone (Sigma-Aldrich, Germany, EU) was dissolved in absolute ethanol to a concentration of 10 mM and diluted in Medium A until treatment concentrations (5 µM, 1 µM, 100 nM and 10 nM) were obtained. In order to keep the environmental conditions as equal as possible, all different culture conditions [Control (Medium A + ethanol), 1 µm, 100 nM and 10 nM rotenone or 5 µM] were performed in the same 4-well plate. Medium was changed every second day until DIV14. On DIV14 sympathetic neurons were fixed using 4 % PFA (Sigma-Aldrich, Germany, EU).

#### Determination of cell loss

**Fig. 1:**
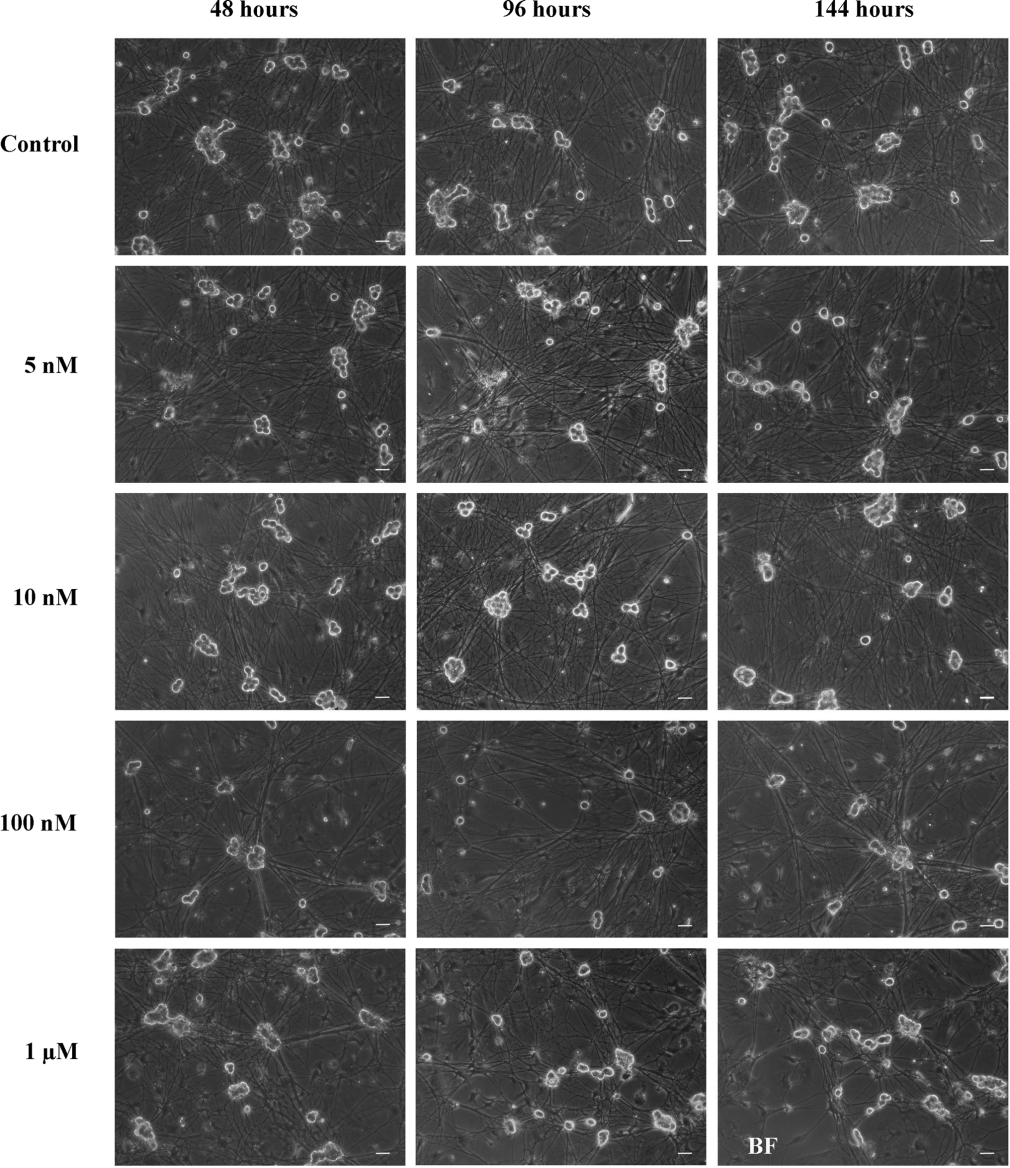
High concentrations of rotenone are needed to induce sympathetic neurite degeneration in vitro. Bright-field microscope images of sympathetic neurons treated with rotenone. *Scale bar* 40 µm. Images show the effect of vehicle (medium with ethanol), 5 nM, 10 nM, 100 nM and 1 µM on sympathetic neurons after 48, 96 and 144 h of rotenone treatment. There is a clear reduction in the neurite density in neurons treated with 100 nM and 1 µM when compared to control neurons or neurons treated with 5 and 10 nM rotenone

In order to quantify the neuronal loss in treated cultures all cells in each well were counted using phase contrast in a bright field microscope. Sympathetic neurons have a characteristic morphology with big somas. This makes it easy to recognize them among other non-neuronal cells (Fig. 1).

#### Adenylate kinase assay

Adenylate kinase is an enzyme present in the media commonly used as an indicator of cell suffering and cell death [17]. Concentration of adenylate kinase in the medium from cultured neurons was measured using the ToxiLight BioAssay (Cambrex, Belgium, EU). 20 µm of medium were placed in a Luminunc™ plate well and 100 µl of the adenylate kinase detection kit were added to the well. After 5 min incubation at RT, luminescence was measured using a microplate luminescence Tecan Genios reader (Tecan, Switzerland) with an integrated read time of 1 s. A black divider was used to avoid cross-talking between wells and the value of a well without medium was used as reference.

#### Immunocytology

Once fixed with 4 % PFA, cultures were washed 4 × 15 min with PBS before starting the immunostaining procedures. Cells were then incubated in blocking solution (BS) [0.4 % Triton X-100 (Thermo Scientific, USA) in PBS, 5 % donkey serum (Jackson Immunoresearch Laboratories, USA)] for 1 h at RT followed by overnight incubation at 4 °C with sheep anti-TH (1:1,000, Pel-Freez, USA), rabbit anti-alpha-synuclein (1:400, Santa-Cruz, USA) and chicken anti-βIII-tubulin (1:500, Novus Biologicals, USA) primary antibodies in BS. On the next day cultures were washed 4 × 15 min with PBS and incubated for 1.5 h at RT using donkey Alexa^®^ 594 anti-sheep and Alexa^®^ 488 anti-rabbit (Invitrogen, USA) or donkey 647 anti-chicken (1:200, Jackson Immunoresearch Laboratories, USA) secondary antibodies in BS. After incubation, secondary antibodies were washed 4 × 15 min with PBS and cells were mounted using Vectashield^®^ mounting medium containing 4,6-diamidino-2-phenylindole (DAPI) (Vector Laboratories, USA).

#### SDS-PAGE and western blotting

Before protein extraction, the number of total sympathetic cells per well were counted. Cultured cells were then wash twice with PBS and lysed in ice-cold lysis buffer [60 mM Tris–HCl (pH 6.8), 1 mM Na_3_VO_4_, 1 % SDS]. Cell debris were removed by centrifugation (15,000 rpm/15 min). Aliquots of the supernatants were quantified using the Qubit™ fluorometric quantification method (Life Technologies, USA) and subjected to SDS-PAGE. Proteins were then transferred onto a nitrocellulose membrane, blocked in milk powder, and membranes were probed for alpha-synuclein (1:400, Santa Cruz Biotech, USA), TH (1:500, Chemicon, USA) or GAPDH (1:2,000, Millipore, USA). Images were then processed using the open access and ImageJ based program FIJI (http://www.fiji.sc), where only minor adjustment of the brightness and contrast were performed.

#### Flow cytometry

Sympathetic neurons were detached from the dish using 1 mg/ml collagenase I (Worthington, USA) and transferred to a 1.5 ml Eppendorf tube (Eppendorf, Germany) where cells were fixed and permeabilized using Cytofix/Cytoperm Kit (BD Bioscience, USA) according to the manufacturer’s protocol. Neurons were then stained against alpha-synuclein with the same antibodies and concentrations as the ones used for immunocytology. All analyses were performed using a LSRII (BD Bioscience, USA) and data were analyzed using FlowJo software (Treestar, USA).

#### RT-PCR

Cell culture was washed with PBS and cell content was extracted using RNAprotect Cell Reagent (Qiagen: 1038674) and PBS (6:1 dilution). Isolation of RNA was performed using the RNeasy Mini Kit from Qiagen (Cat.: 74106). Concentration of RNA was measured using the Qubit Fluorometer (Invitrogen, Germany) and the Quant-iT RNA-Assay Kit (Invitrogen, Germany). RNA was then stored at −80 °C.

qPCR was performed using the VILO cDNA Synthesis Kit (Invitrogen, Germany) following manufacturer’s instructions. qPCR was run as follows one cycle at 95 °C for 10 min, 40 cycles at 95 °C for 15 s and one last cycle for 1 min at 60 °C. We used primers for alpha-synuclein (Qiagen: PPM25867E–200) and GAPDH (Qiagen: PPM02946E–200) sequences. Water and control (treated) samples were treated the same way as the treatment samples avoiding the addition of the Superscript EnzymMix in the RNA to DNA transcription to identify DNA contamination. GAPDH was used as housekeeping RNA. Data was interpreted using the $2^{ - \Delta \Delta C_{\text{t}} }$-method.

### Statistical analysis

Western-blot and image analysis data from in vivo experiments were analyzed using an unpaired *t* test. Alpha-synuclein expression data was analyzed using an unpaired one-way ANOVA test and a Bonferroni’s post hoc analysis to compare all treatments to vehicle. Significance was set at *P* < 0.05.

## Results

### Oral rotenone treatment induces enteric sympathetic denervation in C57BL6 mice

Many PD patients show gastrointestinal alterations prior to the appearance of motor symptoms and a degeneration of the autonomic nervous system (i.e. sympathetic denervation of the heart). We have previously shown that oral rotenone treatment induces gastrointestinal alterations as shown by the 1-h stool collection test. In this study, we also observed a reduction in the amount of stool per hour starting after 2 months of treatment when no motor symptoms could be detected (see Supplementary Figure 1). This reduction can be explained by alterations in the ENS or its autonomic innervation. Therefore, we investigated the effect of orally administered rotenone on the sympathetic innervation of the ENS.

**Fig. 2:**
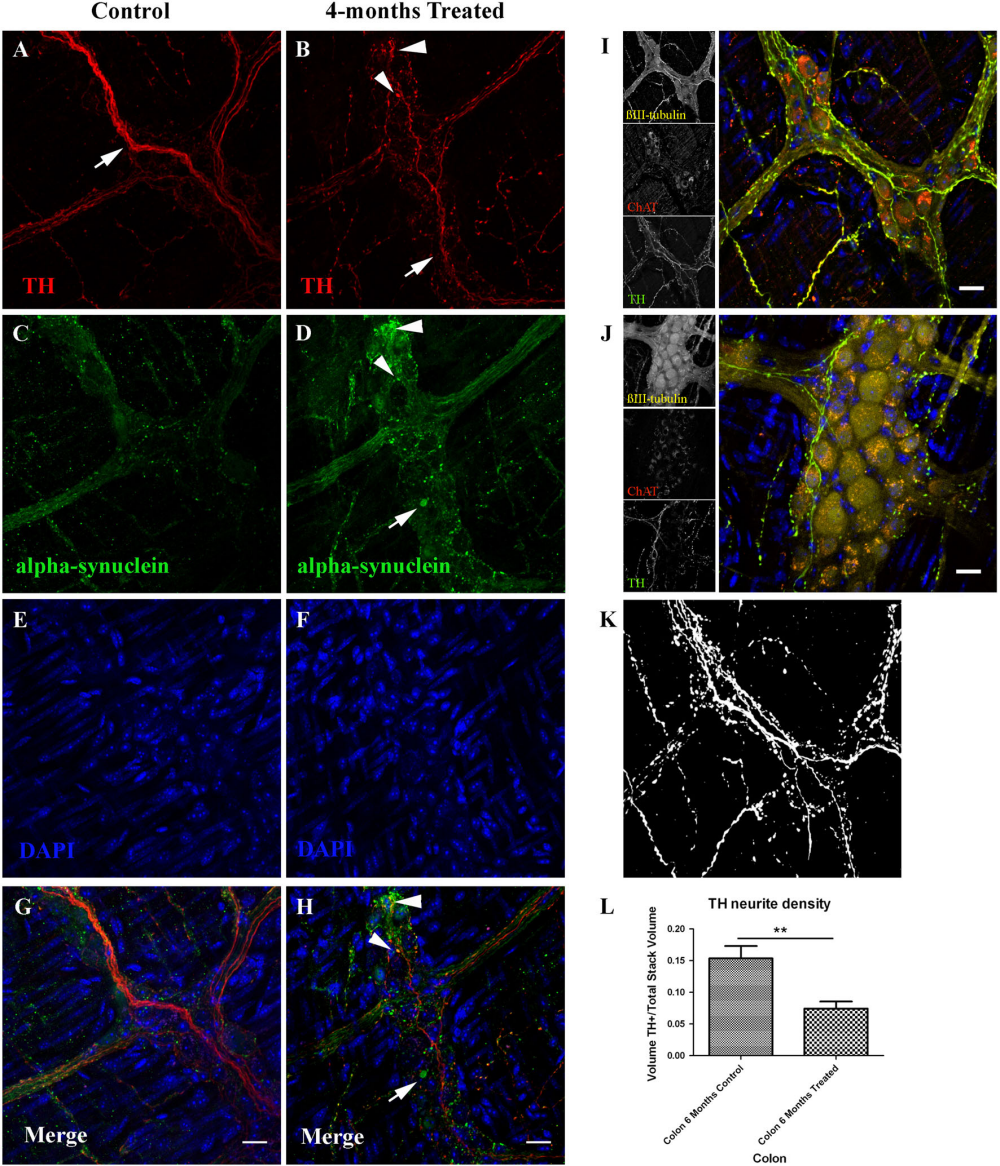
Rotenone ingestion induces enteric sympathetic denervation and alpha-synuclein accumulation. **a**–**h** Whole-mount immunostainings of the colonic Meissner plexus and its sympathetic innervation with antibodies against TH (*red*) and alpha-synuclein (*green*), nuclei were stained with DAPI (*blue*). Treated mice (**b**, **d**, **f**, **h**) show alterations in the amount and morphology of sympathetic neurites (*arrow* in **b**) and a generalized increase in the alpha-synuclein signal and LB-like inclusions (*arrow* in **d**) when compared to control mice (*arrow* in **a** and images in **a**, **c**, **e**, **g**). Some of the alpha-synuclein accumulations co-localized with TH (*arrowheads* in **b**, **d**, **h**). Images in **i** and **j** show whole-mount immunostainings of the colonic Meissner plexus and its sympathetic innervation using antibodies against TH (*green*), ChAT (*red*) and βIII-tubulin (*yellow*), nuclei were stained with DAPI (blue). **k** Segmentation of the TH channel in **i**. Sympathetic neurite density was determined by the volume of TH* positive voxels in the stack divided by the total amount of voxels in the stack. Graphic in **l** shows the results of this quantification analyzed with an Student *t* test. *Error bars* shown the SEM. *Asterisks* shows a significant difference with *P* < 0.01. *Scale bar* in all images represent 20 µm

Immunostaining against alpha-synuclein and TH on colonic whole-mounts showed an increased alpha-synuclein signal with small and big intracellular alpha-synuclein inclusions (Fig. 2a–h), as previously described, and a reduction in the number of TH^+^ sympathetic neurites. Some of the small alpha-synuclein inclusions co-localized with TH within sympathetic neurites. In order to quantify the reduction in the sympathetic innervation, we determined the density of sympathetic neurites innervating the colon. Confocal 3D stacks from whole-mount immunostainings of the colonic muscular layer stained against βIII-tubulin, TH and ChAT were analyzed using image analysis with the help of FIJI software. Our results show a reduction in the density of TH^+^ neurites, determined as the ratio between TH^+^ voxels/total voxels, in rotenone treated mice when compared to controls (*P* < 0.01) (Fig. 2i–l).

### Rotenone treatment induces neurite degradation and alpha-synuclein Lewy-body-like accumulations in sympathetic primary cell cultures

**Fig. 3:**
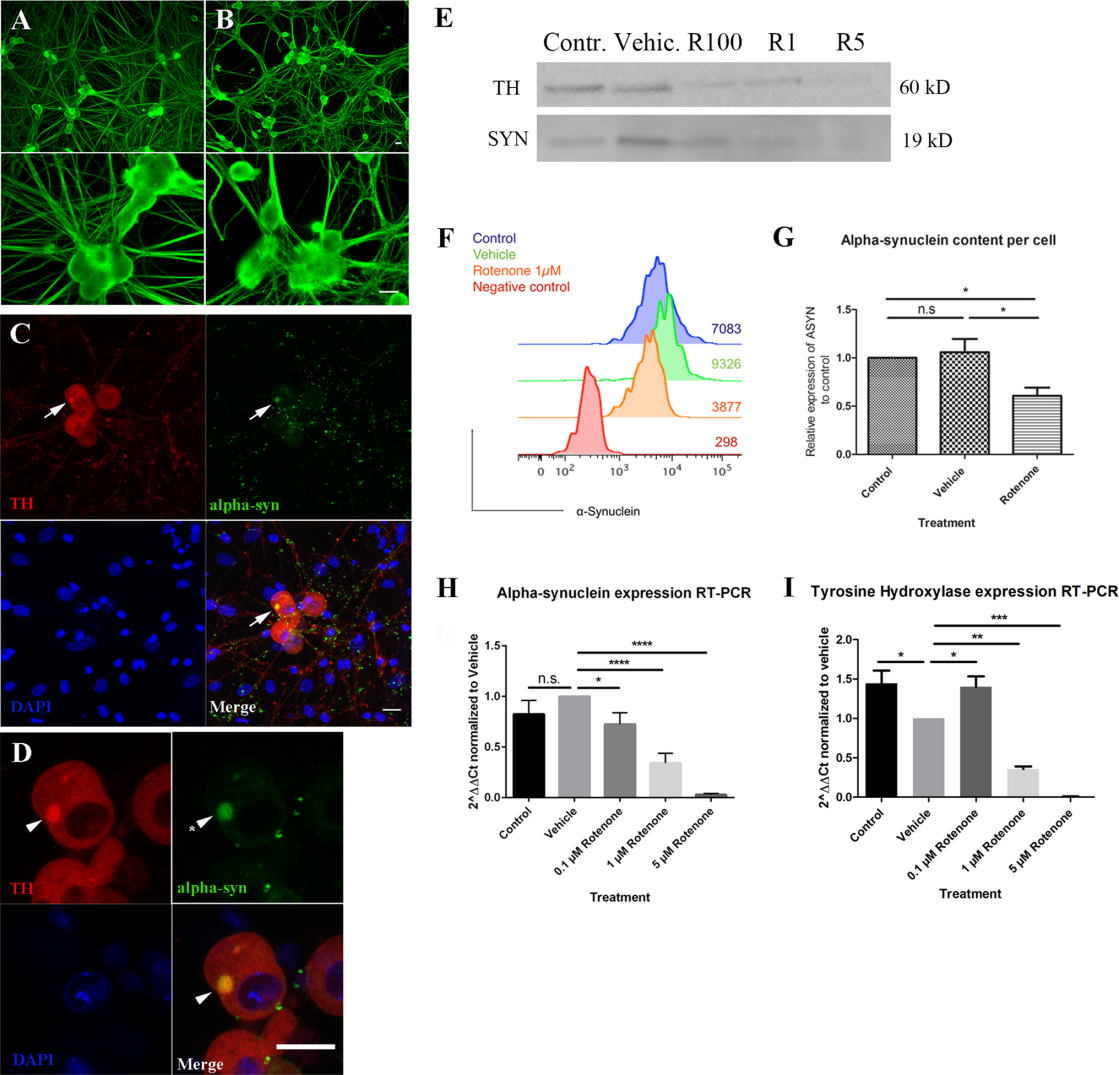
Rotenone treatment on sympathetic neurons in vitro mimics the alterations observed in vivo and down-regulates the expression of alpha-synuclein. **a**–**d** Immunostaining of sympathetic neurons in vitro using antibodies against βIII-tubulin (*green* in **a**), TH (*red* in **c**, **d**) and alpha-synuclein (*green* in **c**, **d**). *Scale bar* 20 µm. Control neurons in **a** show much denser and straight neurites when compared to 1 µM rotenone treated neurons (**b**). Rotenone treatment also induces the appearance of LB-like alpha-synuclein inclusions co-localizing with TH (*arrows* in **c**). Images in **d** show a magnification of the images in **c**, a closer look to these LB-like structures show filamentous projections around the inclusion (*arrowheads* and *asterisk* in **d**). Western-blot (**e**) and FACS analysis (**f**) show a reduced amount of alpha-synuclein in rotenone treated neurons (R100: 100 nM, R1: 1 µM, R5: 5 µM) when compared to control (Contr.) and ethanol treated (Vehicle) neurons. **g** Graphic showing the quantification of FACS analysis (*n* = 3). *Error bars* show SEM, *NS* non-significant. *P < 0.05. Graphic showing the results of a RT-PCR for alpha-synuclein expression (**h**, **i**) RT-PCR showed that rotenone treatment induced the down-regulation of alpha-synuclein (**h**) and tyrosine hydroxylase (**i**) expression. *Error bars* show SEM, *NS* non-significant. *P < 0.05, **P < 0.01, ***P < 0.001

To corroborate our in vivo observations, we set to characterize the effect of rotenone on sympathetic neurons in vitro. Therefore we analyzed the effect of rotenone on the cell morphology and on the distribution/amount of alpha-synuclein in sympathetic neurons in vitro. As mentioned above, using bright-field imaging throughout the treatment, we were able to observe neurite degeneration in rotenone treated neurons (Fig. 1). The degree of neurite impairment/loss was directly proportional to the concentration of rotenone. In line with these results, neurite degeneration could be confirmed by co-immunostaining of βIII-tubulin and TH (Fig. 3a, b). Some accumulations of βIII-tubulin could be observed inside neurites, which gave the neurites a buttoned appearance. Immunostaining against alpha-synuclein showed a difference in the distribution of alpha-synuclein within neurons. Whereas in control sympathetic neurons alpha-synuclein is distributed more uniformly, in rotenone treated neurons alpha-synuclein tends to have a punctate pattern with bigger alpha-synuclein Lewy body-like accumulations in some of the neurons (Fig. 3c). Remarkably, these inclusions were also positive for TH. Imaging using higher magnifications revealed a fibrillar pattern on the edges of the inclusions resembling the one observed in human LB (Fig. 3d).

### Rotenone treatment down-regulates alpha-synuclein expression

In order to further investigate whether the punctate pattern of alpha-synuclein within sympathetic neurons in vitro was due to an increase in alpha-synuclein protein levels, we quantified the amount of alpha-synuclein within cells by Western-blot and flow cytometry. ASYN protein content was found to be reduced in neurons upon rotenone treatment (Fig. 3e). To confirm this finding, we measured the amount of ASYN inside the neurons using flow cytometry. In mixed cell populations as primary neuronal cultures, with contamination from non-neuronal cells, FACS analysis is better suited than Western-blot analysis, as the total alpha-synuclein-associated fluorescent signal can be specifically assigned to TH^+^ sympathetic neurons. Consistently, our results show that the total amount of alpha-synuclein within sympathetic neurons decreased with rotenone treatment (Fig. 3f, g). This reduction in alpha-synuclein was parallel to a reduction in the amount of TH (Fig. 3e).

To investigate if the reduced alpha-synuclein expression upon rotenone treatment is due to a defect in transcription, we measured by RT-PCR the amount of alpha-synuclein mRNA between control, vehicle (containing ethanol at the concentration present with 5 µM rotenone), 0.1, 1 and 5 µM rotenone treated neurons. Indeed, our results show that rotenone treatment induced a down-regulation of alpha-synuclein expression in sympathetic neurons (Fig. 3h). This decrease in the expression of alpha-synuclein was dependent on the concentration of rotenone as cultured neurons cells exposed to various concentrations of rotenone gradually decreased they expression of alpha-synuclein. When compared to vehicle, alpha-synuclein expression under 0.1 µM (0.725 ± 0.065, *P* < 0.05), 1 µM (0.342 ± 0.055, *P* < 0.0001) and 5 µM (0.028 ± 0.006, *P* < 0.0001) rotenone treatment was significantly decreased. Importantly, we did not observe any significant difference between vehicle and control medium (0.82 ± 0.179; *P* > 0.05). A similar reduction was observed in the amount of TH mRNA between control and rotenone treated neurons (Fig. 3i). Interestingly, in the case of TH we observed lower amounts of TH mRNA in vehicle-treated when compared to control and 0.1 µM rotenone, without any statistical differences between the control and 0.1 µM rotenone.

### Sympathetic neurons have an increased resistance to rotenone toxicity when compared to dopaminergic neurons in vitro

It has been postulated that the progression pattern observed in PD patients could be explained by the systemic effect of environmental toxins on the nervous system [18–20]. If this were to be true, the pattern observed would be due to different neuronal sensitivity to systemic intoxication. In our previous study, we showed that orally administered rotenone could reproduce this pattern acting locally on the ENS [21]. However, as our detection limit was 10 ng/ml, the possibility that the pattern observed could be due to the systemic effect of rotenone remained. Therefore, in order to test this hypothesis and further validate the ENS spreading hypothesis, we compared the toxicity of sympathetic and dopaminergic neurons in vitro.

First we cultured sympathetic neurons from C56JBl6 mice and treated them with increasing concentration of rotenone reaching doses up to 100 times higher than those reported to be already extremely toxic for dopaminergic neurons [19] (i.e. 5 nM, 10 nM, 100 nM and 1 µM rotenone) for 6 days. The results showed that sympathetic neurons treated with 5 nM to 1 µM rotenone were able to survive for this time, being mild neurite degeneration the only observable morphological change at a concentration of 1 µM (see Figs. 1, 3). Based on these results we decided to modify the range of rotenone concentrations used, we treated sympathetic and dopaminergic neurons either with control, vehicle with the highest final ethanol concentration used (i.e. the one to treat neurons with 5 µM rotenone) or with 100 nM, 1 µM and 5 µM of rotenone for 10 days using the adenylate kinase cell death assay and the number of remaining neurons after treatment as parameters to determine rotenone toxicity. Additionally, we also performed this experiments in NMRI mice to avoid any interference of the genetic background on the results.

**Fig. 4:**
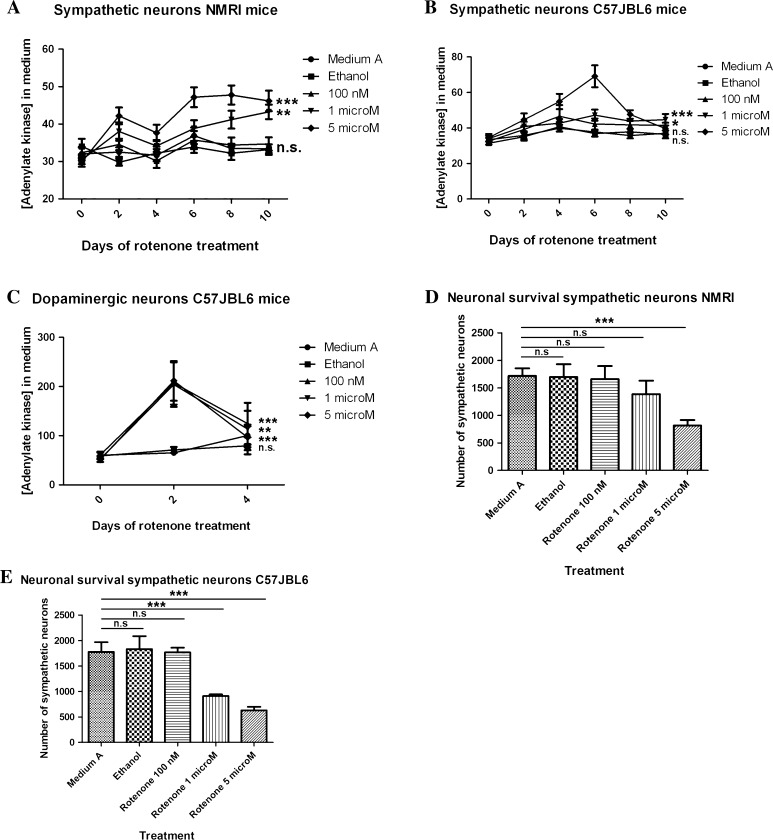
Sympathetic neurons are more resistant to rotenone than dopaminergic neurons. Graphics in **a**–**c** show the concentration of adenylate kinase in the media of NMRI and C57JBL6 sympathetic (**a**, **b**, respectively) and dopaminergic (**c**) neurons treated with control medium (Medium A), vehicle (ethanol) and 100 nM, 1 µM or 5 µM rotenone. Sympathetic neurons need both higher doses and longer treatment times to start dying (shown as an increase in adenylate kinase). *Error* *bar* show SEM, *NS* non-significant. *P < 0.05, **P < 0.01, ***P < 0.0001. Graphics in **d** and **e** show sympathetic neuronal survival upon rotenone treatment after 10 days of rotenone treatment. *Error bar* show SEM, *NS* non-significant. *P < 0.05, **P < 0.01, ***P < 0.0001

The results show a significant increased resistance of sympathetic neurons to rotenone treatment when compared to dopaminergic neurons (Fig. 4a–d). Remarkably, we only observed sympathetic neural death when using rotenone concentrations above 1 µM in C57JBL6 mice (Fig. 4e) and above 5 µM in NMRI mice (Fig. 4d). This correlated well with increases in adenylate kinase values observed both in NMRI mice (Fig. 4a) and C57JBL6 mice (Fig. 4b). In addition while there were still sympathetic neurons alive at the end of the treatment, all rotenone-treated dopaminergic neurons were dead between the second and fourth day of treatment in all rotenone concentrations used. Concentration of adenylate kinase in these cultures also correlated well showing a sudden peak on the second day of treatment that could not be observed on the fourth day (Fig. 4c), suggesting that at this time-point all neuronal cells were dead. This agrees with already published data regarding rotenone toxicity on dopaminergic neurons [19].

## Discussion

Non-motor symptoms in Parkinson’s disease are widely spread among patients. Their prevalence varies depending on the type of symptom but, unlike motor symptoms, they are not always observed in PD patients [22–28]. According to Braak et al.’s [1] pathological staging, most of the nervous structures whose impairment is thought to give raise to non-motor symptoms (e.g. enteric, sympathetic or parasympathetic systems) are affected in very early stages of the disease showing PD-pathology in the form of LB and LN. In this study we show that rotenone treatment induces alpha-synuclein accumulation and the degeneration of the sympathetic innervation of the gut in rotenone treated mice. These alterations are correlated with changes in the motility of the gut.

We also characterized the effect of rotenone on sympathetic neurons in vitro. The degenerative process of the sympathetic ganglia has been observed in PD and Lewy body disease patients and classified within three stages in which neurite loss precedes neuronal loss [11, 12]. In the early stage, a few LBs are observed in the ganglia but the number of neurons and immunoreactivity for TH is well preserved. In the middle stage, many LBs are found in the ganglia and the number of neurons appears to be normal with H&E staining. However, a significant number of neuronal somata (about 20–30 % of neurons) are TH-immunonegative. In the advanced stage, there is apparent neuronal loss in the ganglia and the number of LBs is decreased compared with the middle stage. Interestingly, we could observe similar alterations in vitro by treating sympathetic neurons with different concentrations of rotenone. We observed neurite degeneration and LB-like alpha-synuclein inclusions that were also positive for TH. These alterations could be observed with rotenone concentrations higher than 100 µM and preceded sympathetic neuronal death in the case of the highest concentration of rotenone (5 µM). Additionally, we also observed a decrease in the amount of TH within sympathetic cells.

Previous studies have shown both an up-regulation and a down-regulation of alpha-synuclein expression upon exposure to different toxins [29–31]. It is still unclear whether the aggregation of alpha-synuclein is induced by an up-regulation of its expression or by post-translational modifications. We analyzed the effect of rotenone treatment on alpha-synuclein expression. Despite the appearance of alpha-synuclein inclusions, our results show a down regulation of alpha-synuclein expression upon rotenone treatment. It can be argued, that this reduction in alpha-synuclein expression is linked to neuronal loss, as also the expression of TH inside sympathetic cells was reduced. However, we quantified the total amount of sympathetic neurons in every treatment before protein extraction and we did not observed any sympathetic neuronal loss after 100 nM rotenone-treatment and only a mild neuronal loss after treatment with 1 µm rotenone (see results Fig. 4). Interestingly, in both cases we did observe significant neurite impairment with a reduction of neurite density. These results led us to speculate that the down-regulation of alpha-synuclein expression and TH levels could be linked to the neurite degeneration. Alpha-synuclein is a presynaptic protein and TH is an enzyme needed to produce noradrenaline also localized to the synaptic terminals. Therefore, the disappearance of the neurites could induce a feedback leading to the down-regulation of alpha-synuclein and TH expression. Altogether these results suggest that the appearance of ASYN accumulations within sympathetic neurons is not due to an increase in its expression and is independent of the total alpha-synuclein content. Thus, suggesting that other processes such as alpha-synuclein post-translational modifications may be responsible for the increase in alpha-synuclein to alpha-synuclein interactions.

Two hypothesis have been postulated to explain the appearance and progression of PD pathology in patients: (1) a double action (peripheral and central) of a systemic noxa and (2) the enteric/olfactory spreading hypothesis, that postulates that toxins or infectious agents acting on the ENS and the OB could trigger the appearance of the pathology and its progression into and throughout the CNS. As PD has been related to the exposure to pesticides [32, 33], some authors suggest that the progression pattern observed in PD patients could be explained by the systemic effect of environmental toxins on the nervous system [18–20] leading to the first hypothesis. If we apply this hypothesis to our model, the pathology progression pattern that we observe would be a consequence of neuronal differences in the sensitivity to Complex I inhibition. Therefore, sympathetic neurons should be more sensitive to rotenone treatment than dopaminergic neurons. In order to test the first hypothesis, we compared the effect of different concentrations of rotenone on sympathetic and dopaminergic neurons in vitro. Our results clearly show that dopaminergic neurons are up to 100 times more sensitive to rotenone than sympathetic neurons, suggesting that the pathology progression observed in oral rotenone treated mice is not due to a systemic effect of the substance. In the last years, different studies have shown that injected or locally produced ASYN can spread from one region to another of the nervous system [34–36]. More recently, a study showed that full and partial vagotomy reduced the risk of developing Parkinson’s disease [37]. Thus supporting the spreading hypothesis.

Although clinical studies are needed, PET–CT studies using meta-iodobenzylguanidine (MIBG) or similar radiotracers could be used to detect variations in the sympathetic neurite density of the gut. Our results suggest that the sympathetic denervation of the ENS is an early event in the progression of PD and has the potential to be used as biomarker for the diagnosis of the disease in its initial steps.

## Electronic supplementary material

Below is the link to the electronic supplementary material.

**Supplementary Figure 1: Rotenone treatment reduces intestine motility** Bar graph showing the weight of the stool collected per animal within 1 h at time points 1, 2, 3 and 4 months after treatment. Error bar show s.e.m., n.s. is non-significant. * is *P*<0.05. ** is *P*<0.01 (TIFF 43 kb)
